# Human Papillomavirus and Cystic Node Metastasis in Oropharyngeal Cancer and Cancer of Unknown Primary Origin

**DOI:** 10.1371/journal.pone.0095364

**Published:** 2014-04-21

**Authors:** Toshimichi Yasui, Eiichi Morii, Yoshifumi Yamamoto, Tadashi Yoshii, Yukinori Takenaka, Susumu Nakahara, Takeshi Todo, Hidenori Inohara

**Affiliations:** 1 Department of Otorhinolaryngology-Head and Neck Surgery, Osaka University Faculty of Medicine, Suita, Osaka, Japan; 2 Department of Pathology, Osaka University Faculty of Medicine, Suita, Osaka, Japan; 3 Department of Radiation Biology and Medical Genetics, Osaka University Faculty of Medicine, Suita, Osaka, Japan; Penn State University School of Medicine, United States of America

## Abstract

The clinical significance of human papillomavirus (HPV) in neck node metastasis from cancer of unknown primary (CUP) is not well established. We aimed to address the relationship of HPV status between node metastasis and the primary tumor, and also the relevance of HPV status regarding radiographically detected cystic node metastasis in head and neck squamous cell carcinoma (HNSCC) and CUP. HPV DNA was examined in 68 matched pairs of node metastasis and primary tumor, and in node metastasis from 27 CUPs. In surgically treated CUPs, p16 was examined immunohistochemically. When tonsillectomy proved occult tonsillar cancer in CUP, HPV DNA and p16 were also examined in the occult primary. Cystic node metastasis on contrast-enhanced computed tomography scans was correlated with the primary site and HPV status in another series of 255 HNSCCs and CUPs with known HPV status. Node metastasis was HPV-positive in 19/37 (51%) oropharyngeal SCCs (OPSCCs) and 10/27 (37%) CUPs, but not in non-OPSCCs. Fluid was collected from cystic node metastasis using fine needle aspiration in two OPSCCs and one CUP, and all fluid collections were HPV-positive. HPV status, including the presence of HPV DNA, genotype, and physical status, as well as the expression pattern of p16 were consistent between node metastasis and primary or occult primary tumor. Occult tonsillar cancer was found more frequently in p16-positive CUP than in p16-negative CUP (odds ratio (OR), 39.0; 95% confidence interval (CI), 1.4–377.8; P = 0.02). Radiographically, cystic node metastasis was specific to OPSCC and CUP, and was associated with HPV positivity relative to necrotic or solid node metastasis (OR, 6.2; 95% CI, 1.2–45.7; P = 0.03). In conclusion, HPV status remains unchanged after metastasis. The occult primary of HPV-positive CUP is most probably localized in the oropharynx. HPV status determined from fine needle aspirates facilitates the diagnosis of cystic node metastasis.

## Introduction

Cervical lymph node metastasis from cancer of unknown primary (CUP) is a rare clinical entity and currently accounts for no more than 3% of head and neck squamous cell carcinoma (HNSCC) [1]. In many cases following its initial presentation as CUP, the primary site is unveiled predominantly in the oropharynx, particularly in the palatine tonsil and the base of the tongue [2]. Oropharyngeal squamous cell carcinoma (OPSCC) is etiologically classified into two distinct subtypes; one is tobacco related, while the other is caused by human papillomavirus (HPV) infection [3], [4]. Overexpression of p16 serves as a surrogate marker of HPV infection, although a subset of p16-positive tumors are HPV-negative [4]. HPV-positive and/or p16-positive OPSCC preferentially arises in the palatine tonsil and the base of the tongue [4], suggesting the relevance of HPV to CUP.

HPV prevalence in node metastasis from HNSCC has been well studied; it has been shown that HPV-positive node metastasis is specific to OPSCC [5], [6], whereas the relationship of HPV status between the primary tumor and its corresponding node metastasis is not well established. Although a series of studies have shown good concordance of HPV status between the primary tumor and node metastasis, the sample size of each study was small, and HPV physical status was not addressed [7]–[9]. HPV prevalence in node metastasis from CUP has been reported to vary from 28 to 92%, depending on the definition of CUP [8]–[11], whereas the relationship of HPV status between the node metastasis and its corresponding occult primary tumor has not been established.

The association of OPSCC with histologically identified cystic node metastasis is well known [12], while the majority of occult primaries in CUP with histologically identified cystic node metastasis are localized in the palatine tonsil or the base of the tongue [13]; this suggests the involvement of HPV infection in the formation of cystic node metastasis. Goldenberg et al. analyzed HPV status in dissected tissues from the necks of OPSCC and CUP patients, and found that 87% (13/15) of cystic metastatic nodes were HPV-positive [14]. In another study, it has been reported that a branchial cleft cyst often proves to be cystic node metastasis after surgical intervention followed by histopathological examination [15]. The reason for this is that both cystic node metastasis and a branchial cleft cyst present radiographically as lateral cervical cysts, and that sensitivity regarding the detection of SCC cells in cystic fluid by means of cytopathological examination is poor. Accordingly, a nonsurgical differential diagnosis between these two entities in terms of HPV status is of clinical interest. The differential diagnosis requires not only establishing an association between HPV status and radiographically identified cystic node metastasis, but also the development of HPV analysis of cystic fluid collected by fine needle aspiration (FNA).

We designed the present study to better understand the clinical significance of HPV status regarding node metastasis from CUP. To this end we investigated HPV status, including the presence of HPV DNA, genotype, and physical status, in matched pairs of primary tumor and node metastasis in a large series of HNSCC, and evaluated the relationship between the primary tumor and its node metastasis regarding HPV status. We further investigated not only HPV prevalence in node metastasis from CUP, but also the relationship between the node metastasis and its corresponding occult tonsillar cancer (proved after tonsillectomy) with regard to HPV status and p16 expression pattern. In addition, we studied the relationship between radiographically detected cystic node metastasis and the primary site and HPV status, with an emphasis on HPV detection in cystic fluid.

## Materials and Methods

### Ethics Statement

The protocol of the present study was approved by the Institutional Review Board of Osaka University in December 2004. All patients provided written informed consent.

### Patients

The HPV status of the primary tumor and its corresponding node metastasis, as well as the HPV status of node metastasis from CUP, were addressed in both retrospective and prospective settings. In the retrospective setting, formalin-fixed paraffin-embedded (FFPE) specimens from 28 matched pairs of primary and node metastasis and from 17 CUPs were enrolled. All of the tumors were surgically excised between January 2005 and March 2010 without any prior treatment, and were histopathologically diagnosed as squamous cell carcinoma. In the prospective setting, a total of 53 suspicious node-positive HNSCCs and CUPs were enrolled. Biopsy specimens of suspicious primary tumors and FNA specimens of suspicious node metastasis were obtained from outpatients between April 2010 and September 2012. Biopsy specimens and FNA specimens were stored in RNAlater RNA stabilization reagent (QIAGEN Inc., Valencia, CA, USA) and buffer ATL (QIAamp Mini Kit: QIAGEN Inc.), respectively. When fluid collection was undertaken using FNA, its centrifugate was stored as detailed above. Only a single cycle of FNA was carried out for the HPV analysis, whereas FNA was repeated as necessary for cytopathological analysis. When tonsillectomy revealed an occult primary tumor in CUP patients, the FFPE specimen of the occult primary was analyzed for HPV DNA and p16. Expression of p16 was also examined in surgically excised node metastasis from CUP, irrespective of the settings. For radiographic analysis, a total of 255 HNSCC and CUP patients, who had undergone a pretreatment contrast-enhanced CT scan and had been proven pathologically to be node positive, were enrolled. This population included the retrospective series of 45 HNSCCs/CUPs, the prospective series of 50 HNSCCs/CUPs, and the additional series of 160 HNSCCs. The HPV status of the additional series had been evaluated using FFPE biopsy specimens from the primary tumor [16]. Diagnosis of CUP was made after thorough workup failed to detect a primary tumor despite pathologically proven node metastasis. Workup included the following: medical history; physical examination; neck CT scan and/or MRI; pharyngolaryngeal endoscopy; upper gastrointestinal endoscopy; mucosal biopsy of the oropharynx and nasopharynx; and ^18^F-fluorodeoxyglucose positron emission tomography (FDG-PET) or FDG-PET/CT. Tonsillectomy was not included in the criteria because in principle tonsillectomy was performed simultaneously with neck dissection when the tumor was resectable.

### Detection and typing of high-risk HPV DNA and assessment of HPV16 physical status

DNA was extracted from FFPE specimens using the DNeasy tissue kit (QIAGEN Inc.), and from fresh specimens using the QIAamp Mini Kit (QIAGEN Inc.). The presence of HPV DNA was screened for by means of a nested polymerase chain reaction (PCR) using the PGMY09/11 primer set (for primary PCR) and the GP5+/6+ primer set (for secondary PCR) as previously reported [17]. The secondary PCR products were purified and sequenced directly using a 3100 Genetic Analyzer (Applied Biosystems, Foster City, CA, USA). Typing was achieved by comparing the sequence with those of known HPV types using the NCBI BLAST program (http://blast.ncbi.nlm.nih.gov/Blast.cgi). The physical status of HPV16 was addressed according to the ratio of *E2* to *E6* copy numbers, which was determined by means of real-time PCR amplification of the *E2* and *E6* open reading frames as previously reported [18]. The cervical cancer cell line Caski was used as a positive control [19].

### Immunohistochemistry of p16

Immunohistochemical analysis of p16 was carried out using 4-µm FFPE tissue sections with an appropriate positive control. Following antigen retrieval using a Pascal pressurized heating chamber (DAKO, Glostrup, Denmark), the sections were incubated with anti-p16 antibody at a dilution of 1∶500 (clone LC8: Santa Cruz Biotechnology, Inc., Dallas, TA, USA). After incubation, anti-p16 antibody was detected using the ChemMate EnVision kit (DAKO) and visualized by means of diaminobenzidine as a chromogen. Negative control staining was carried out in the absence of primary antibody. Cases were classified as positive for p16 when 50% or more of cells showed nuclear and cytoplasmic staining.

### Definition of cystic node on contrast-enhanced CT scan

**Figure 1:**
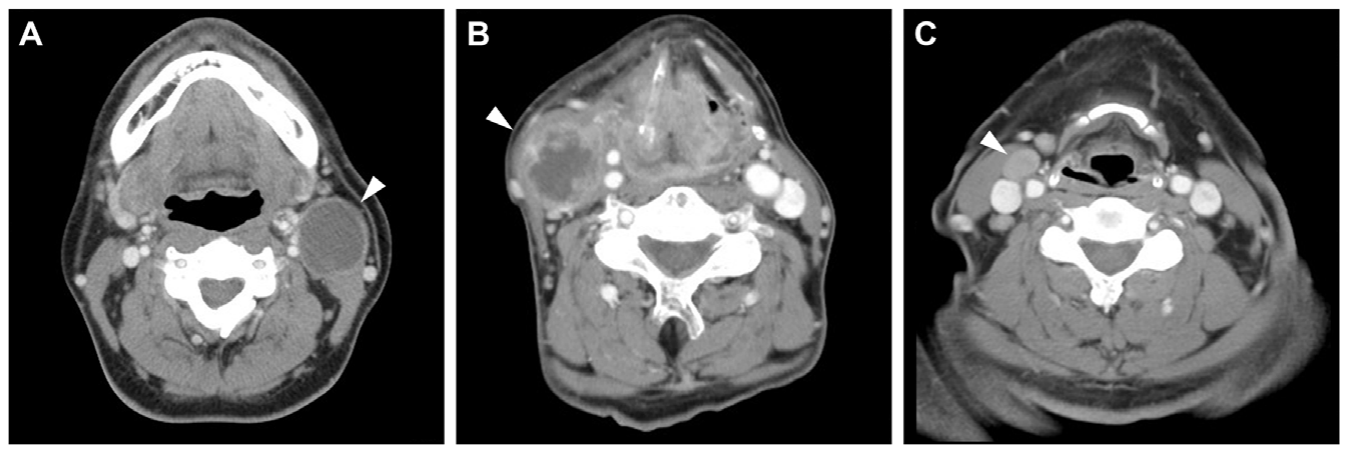
Radiographically identifiable (A) necrotic, (B) solid, and (C) cystic node metastasis defined by axial contrast-enhanced CT scans. Note a contrast-enhancing thin wall and homogeneous low-density content in the cystic node metastasis.

The definition of cystic nodes on contrast-enhanced CT scans was in accordance with the criteria reported by Goldenberg et al. [14]. Nodes with a round or ovoid shape, a thin (<2 mm) enhancing capsule, homogeneous water attenuation and no internal complex that were heterogeneous, or had solid area were classified as cystic (Fig. 1a). Nodes with thicker solid walls and/or heterogeneous, complex central low attenuation were classified as necrotic (Fig. 1b); while nodes with a homogeneous solid content were classified as solid (Fig. 1c).

### Statistical analysis

The symmetry of HPV status and/or p16 expression between the primary tumor and its corresponding node metastasis was examined using the kappa test. The difference in the phenotype of HPV/p16 between node metastasis from CUP (with and without occult tonsillar cancer) was examined using the Fisher-Freeman-Halton exact test. The relationships between occult tonsillar cancer and HPV status and between occult tonsillar cancer and p16 expression were examined by means of univariate analysis using the logistic regression model and the Clopper-Pearson exact test, respectively. Differences in the prevalence of cystic node metastasis according to the primary site were assessed by means of univariate analysis using the logistic regression model or the Clopper-Pearson exact test, as appropriate. The relationship between HPV status and cystic node metastasis was assessed by means of univariate analysis using the logistic regression model. All statistical tests were two tailed. P values of <0.05 were considered as being statistically significant. All data were analyzed using JMP Version 10.02 software (SAS Institute, Cary, NC, USA).

## Results

### Relationship of HPV status between primary and node metastasis in OPSCC

In the retrospective setting, 40% (6/15) of OPSCCs were HPV-positive and 60% (9/15) were HPV-negative, regarding both the primary tumor and its corresponding node metastasis (Table 1). The presence or absence of HPV infection was consistent between the primary tumor and its corresponding node metastasis, indicating the similarity in HPV status between the two (P<0.0001). Of note is the fact that 17% (1/6) of HPV-positive OPSCCs had multiple metastatic nodes, all of which were HPV-positive. Conversely, 33% (3/9) of HPV-negative OPSCCs had multiple metastatic nodes, all of which were HPV-negative. None of the 13 non-OPSCCs (5 hypopharyngeal, 4 laryngeal, and 4 oral cancers) were HPV-positive.

**Table 1:** Relationship between the primary tumor and node metastasis regarding HPV status.

				HPV Status in Node Metastasis				
Setting	HPV Status in Primary			HPV-Negative	(%)	HPV-Positive	(%)	P Value*
Retrospective	Oropharynx	(N = 15)	HPV-negative	9	(60)	0	(0)	<0.0001
			HPV-positive	0	(0)	6	(40)	
	Non-oropharynx	(N = 13)	HPV-negative	13	(100)	0	(0)	
			HPV-positive	0	(0)	0	(0)	
	Unknown	(N = 17)		11	(65)	6	(35)	
Prospective	Oropharynx	(N = 22)	HPV-negative	9	(41)	0	(0)	<0.0001
			HPV-positive	0	(0)	13	(59)	
	Non-oropharynx	(N = 18)	HPV-negative	18	(100)	0	(0)	
			HPV-positive	0	(0)	0	(0)	
	Unknown	(N = 10)		6	(60)	4	(40)	

*Symmetry of HPV status between the primary tumor and its corresponding node metastasis in oropharyngeal cancer was examined using the kappa test.

HPV: human papillomavirus.

As for the prospective setting, PCR analysis was unsuccessful in 6% (3/53) of cases because of poor DNA preparation from FNAs. However, repeated FNAs evidenced malignancy in all three of these cases. Accordingly, the prospective analysis was limited to 50 cases, which were finally diagnosed by thorough clinical workup to be the following cancers: 22 oropharyngeal; 2 nasopharyngeal; 7 hypopharyngeal; 7 laryngeal; 2 oral; and 10 CUPs. In both the primary tumor and its node metastasis, 59% (13/22) of OPSCCs were HPV-positive, whereas 41% (9/22) of OPSCCs and 100% (18/18) of the non-OPSCCs were HPV-negative in both the primary tumor and its node metastasis; again, this indicated the similarity in HPV status between the primary tumor and its node metastasis (P<0.0001; Table 1). Collectively, 51% (19/37) of OPSCCs were HPV-positive, all of which originated in either the palatine tonsil or the base of the tongue. As shown in Tables 2 and 3, both HPV genotype and physical status were consistent between the primary tumor and its node metastasis (P<0.0001). HPV16 accounted for 95% (18/19) of HPV-positive cases and HPV33 was detected in the remaining one case. Complete viral integration was found in 56% (10/18) of the HPV16-positive cases, while the remaining 44% (8/18) of HPV16-positive cases exhibited a mixture of episomal and integrated viral forms.

**Table 2:** Relationship between the primary tumor or occult primary tumor and node metastasis regarding HPV genotype.

			HPV Genotype in Node Metastasis				
HPV Genotype in Primary			HPV16	(%)	HPV33	(%)	P Value*
Oropharynx	(N = 19)	HPV16	18	(95)	0	(0)	<0.0001
		HPV33	0	(0)	1	(5)	
Occult tonsil	(N = 3)	HPV16	3	(100)	0	(0)	

*Symmetry of HPV genotype between the primary tumor and its corresponding node metastasis in oropharyngeal cancer patients was examined using the kappa test.

HPV: human papillomavirus.

**Table 3:** Relationship between the primary or occult primary and node metastasis regarding HPV physical status.

			HPV16 Physical Status in Node Metastasis						
HPV16 Physical Status in Primary			Integrated	(%)	Mixed	(%)	Episomal	(%)	P Value*
Oropharynx	(N = 18)	Integrated	10	(56)	0	(0)	0	(0)	<0.0001
		Mixed	0	(0)	8	(44)	0	(0)	
		Episomal	0	(0)	0	(0)	0	(0)	
Occult tonsil	(N = 3)	Integrated	1	(33)	0	(0)	0	(0)	<0.0001
		Mixed	0	(0)	2	(67)	0	(0)	
		Episomal	0	(0)	0	(0)	0	(0)	

*Symmetry of HPV physical status between the primary tumor and its corresponding node metastasis in oropharyngeal cancer and occult tonsil cancer patients was examined using the kappa test.

HPV: human papillomavirus.

### HPV prevalence in CUP and relationship of HPV status between the occult primary and node metastasis

In the retrospective and prospective settings, 35% (6/17) and 40% (4/10) of CUPs were HPV-positive, respectively, indicating that a total of 37% (10/27) of CUPs were HPV-positive (Table 1). Detailed characteristics of CUPs with respect to HPV are presented in Table S1 for the retrospective setting and in Table S2 for the prospective setting. HPV16 and HPV18 accounted for 80% (8/10) and 20% (2/10) of the 10 HPV-positive cases, respectively. Complete viral integration was found in 63% (5/8) of HPV16-positive cases, while the remaining 38% (3/8) of HPV16-positive cases showed mixed viral forms. It should be noted that fluid was collected by FNA in three cases (2 OPSCCs and 1 CUP) in the prospective setting. All of the three fluid collections were found to be HPV-positive, although repeated FNA cytology failed to detect malignant cells in one of them.

**Figure 2:**
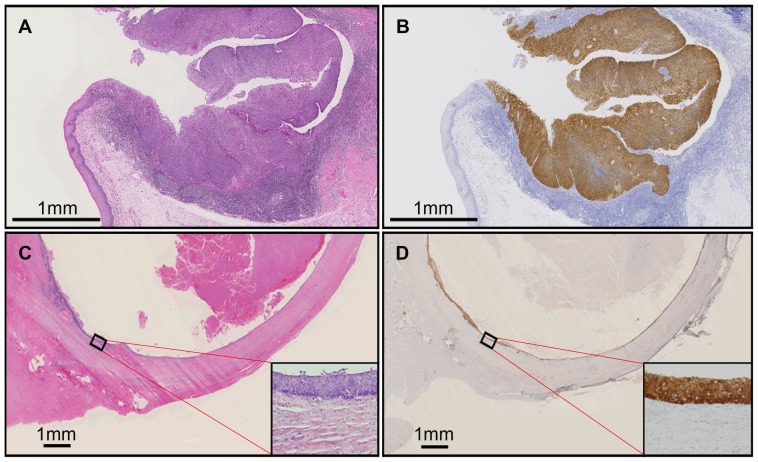
Hematoxylin and eosin staining (A, C) and p16 immunohistochemistry (B, D) of occult tonsillar cancer (A, B) and its corresponding cystic node metastasis (C, D). The scale bar corresponds to 1 mm.

Of 27 patients with CUP, 19 underwent tonsillectomy along with neck dissection, which revealed occult tonsillar cancer in 32% (6/19). The relationship between node metastasis and its corresponding occult tonsillar cancer regarding HPV status and p16 expression are summarized in Table 4. The phenotype of HPV status and p16 expression was consistent between node metastasis and its corresponding occult tonsillar cancer (P = 0.01). Moreover, as shown in Tables 2 and 3, HPV genotype and physical status were consistent between node metastasis and its corresponding occult tonsillar cancer. Fig. 2 depicts hematoxylin and eosin staining and p16 immunohistochemistry of occult tonsillar cancer with cystic node metastasis. The superficial mucosal layer of the tonsil was found to be intact, while cancer cells expressing p16 were prominent in a crypt layer with invasion into the submucosal layer (Fig. 2a and 2b). Cancerous tissue that delineated a thin cystic wall diffusely expressed p16 (Fig. 2c and 2d).

**Table 4:** Relationship between the occult tonsillar primary and node metastasis regarding HPV status and p16 expression.

			Phenotype in Node Metastasis						
Occult Tonsillar Primary		Phenotype in Occult Primary	HPV-Negative/p16-Negative	(%)	HPV-Negative/p16-Positive	(%)	HPV-Positive/p16-Positive	(%)	P Value*
Unproven	(N = 13)		10	(77)	1	(8)	2	(15)	
Proven	(N = 6)	HPV-negative/p16-negative	0	(0)	0	(0)	0	(0)	0.01
		HPV-negative/p16-positive	0	(0)	3	(50)	0	(0)	
		HPV-positive/p16-positive	0	(0)	0	(0)	3	(50)	

*Symmetry of HPV/p16 phenotype between the occult tonsillar primary and node metastasis was examined using the kappa test.

HPV: human papillomavirus.

Of note, 100% (6/6) of the CUPs with unveiled occult tonsillar cancer expressed p16, while 50% (3/6) were HPV-positive. In sharp contrast, only 23% (3/13) and 15% (2/13) of the CUPs without unveiled occult tonsillar cancer were p16-positive and HPV-positive, respectively. The difference in the distribution of HPV/p16 phenotypes was statistically significant between the CUPs, with and without unveiled occult tonsillar cancer (P = 0.004); this finding prompted us to investigate the relationship between occult tonsillar cancer and HPV status or p16 expression in node metastasis from CUP. As shown in Table 5, the possibility that occult tonsillar cancer could be discovered after tonsillectomy was significantly higher when node metastasis from CUP was p16-positive, than when it was p16-negative (odds ratio (OR), 39.0; 95% confidence interval (CI), 1.4–377.8; P = 0.02). Although occult tonsillar cancer was proven more frequently in HPV-positive CUP than in HPV-negative CUP, the difference did not reach statistical significance (P = 0.17)

**Table 5:** Association of occult tonsillar cancer with HPV status and p16 expression in node metastasis from cancer of unknown primary.

		Occult Tonsillar Cancer					
		Proven					
Node Metastasis		No.	%	Unproven (No.)	Odds Ratio	95% CI	P Value*
HPV-negative	(N = 14)	3	21	11	Reference		
HPV-positive	(N = 5)	3	60	2	5.5	0.5–59.1	0.17
p16-negative	(N = 10)	0	0	10	Reference		
p16-positive	(N = 9)	6	67	3	39.0	1.4–377.8	0.02

*Univariate analysis using the logistic regression model and the Clopper-Pearson method were carried out regarding HPV status and p16 expression, respectively.

HPV: human papillomavirus; CI: confidence interval.

### Association of radiographically identified cystic node metastasis with primary tumor site and HPV status

Contrast-enhanced CT scans from a total of 255 patients with node-positive HNSCC or CUP were reviewed (Table 6). None of 146 non-OPSCCs (18 nasopharyngeal, 91 hypopharyngeal, 25 laryngeal, and 12 oral cancers) showed radiographically detected cystic node metastasis, while 6% (5/82) of OPSCCs and 11% (3/27) of CUPs did. All of the five OPSCCs with radiographically detected cystic node metastasis originated in either the palatine tonsil or the base of tongue. The incidence of cystic node metastasis was significantly lower in non-OPSCC than in OPSCC (OR, 0.05; 95% CI, 0.004–0.8; P = 0.03). Of the cases with cystic node metastasis, 38% (3/8) showed multiple nodal metastasis, all of which involved necrotic node metastasis. All of the remaining five cases showed solitary nodal metastasis, which was cystic. Because both HPV infection and cystic node metastasis were specific to OPSCC and CUP, we investigated the association of HPV status with radiographic finding of node metastasis in OPSCC and CUP (Table 7). As compared with necrotic and/or solid node metastasis, cystic node metastasis was more likely to be HPV-positive (33 versus 75%; OR, 6.2; 95% CI, 1.2–45.7; P = 0.03). In turn, the frequency of radiographically identifiable cystic node metastasis was higher in HPV-positive tumor than in HPV-negative tumor (15 versus 3%; OR, 6.2; 95%CI, 1.3–43.8; P = 0.02).

**Table 6:** Association of radiographically identified cystic node metastasis with the primary site.

		Radiographic Finding of Node Metastasis					
		Cystic					
Primary Site		No.	%	Necrotic/Solid (No.)	Odds Ratio	95% CI	P Value*
Oropharynx	(N = 82)	5	6	77	Reference		
Non-oropharynx	(N = 146)	0	0	146	0.05	0.004–0.8	0.03
Unknown	(N = 27)	3	11	24	1.9	0.4–8.9	0.41

*Univariate analysis was carried out regarding the non-oropharynx site using the Clopper-Pearson method and for the unknown primary site using the logistic regression model.

CI: confidence interval.

**Table 7:** Association of HPV status with radiographic finding of node metastasis in oropharyngeal cancer and cancer of unknown primary.

		HPV Positive					
Radiographic Finding of Node Metastasis		No.	%	HPV Negative (No.)	Odds Ratio	95% CI	P Value*
Necrotic/Solid	(N = 101)	33	33	68	Reference		
Cystic	(N = 8)	6	75	2	6.2	1.2–45.7	0.03

*Univariate analysis was carried out using the logistic regression model.

HPV: human papillomavirus; CI: confidence interval.

## Discussion

In the present study, we have shown that HPV-positive node metastasis is specific to OPSCC, especially when OPSCC arises in the palatine tonsil and the base of the tongue. We also demonstrated that HPV status, including the presence of HPV DNA, genotype, and physical status, was consistent between the primary tumor and its corresponding node metastasis. These results suggest that the occult primary tumor of CUP with HPV-positive node metastasis is most probably localized in the oropharynx, particularly in the palatine tonsil or the base of the tongue. Findings regarding our series of CUPs were in accordance with this suggestion. Tonsillectomy revealed occult tonsillar cancer in 60% (3/5) of the CUPs with HPV-positive node metastasis and 67% (6/9) of the CUPs with p16-positive node metastasis, but in none of 10 CUPs with HPV-negative/p16-negative node metastasis. Moreover, we have shown for the first time that HPV status, including the presence of HPV DNA, genotype, and physical status, as well as the expression pattern of p16 are consistent between node metastasis and its corresponding occult tonsillar cancer. Collectively, we have established not only that the HPV status of the tumor remains unchanged after metastasis, but also that the occult primary site of CUP with HPV-related node metastasis exists in the oropharynx.

Given these findings, it is most likely in the case of CUP with HPV-related node metastasis that the occult primary tumor is localized at the base of the tongue, unless occult tonsillar cancer is proved after tonsillectomy. Weiss et al. reported that tonsillectomy and blind biopsy of the base of the tongue unveiled the occult primary tumor in all of the 12 CUPs with HPV-positive node metastasis, and in all of the 11 CUPs with p16-positive node metastasis [8]. In our series, tonsillectomy failed to reveal occult tonsillar cancer in 40% (2/5) of CUPs with HPV-positive node metastasis, and in 33% (3/9) of CUPs with p16-positive node metastasis, in which the occult primary tumor was expected to be localized at the base of the tongue. Furthermore, we found that tonsillectomy was unsuccessful in detecting the occult primary tumor in all of the 10 CUPs with HPV-negative/p16-negative node metastasis. Likewise, Park et al. reported that tonsillectomy and mucosal biopsy were successful in the identification of occult OPSCC in only 7% (2/27) of CUPs with HPV-negative node metastasis and in 14% (4/29) of CUPs with p16-negative node metastasis [10]. It seems that it is difficult to identify the occult primary tumor using tonsillectomy when node metastasis from CUP is HPV-negative/p16-negative.

According to a systematic review of HPV types in HNSCC, the prevalence of HPV18 in OPSCC was low; HPV18 accounted for only 9 of 909 (1.0%) HPV-positive OPSCC cases [20]. Moreover, the vast majority (>95%) of HPV18-positive HNSCCs were not OPSCCs. In contrast, HPV18 accounted for 2 of 10 (20%) HPV-positive CUPs in our series; unfortunately, both of the patients with HPV18-positive CUPs did not undergo tonsillectomy (Table S1 and S2). It remains unclear as to whether or not the occult primary site in these two CUPs was the tonsil. Given the aforementioned finding that non-oropharyngeal SCC form the vast majority of HPV18-positive HNSCC cases, it is likely that the occult primary of HPV18-positive CUP exists at sites other than the oropharynx. This is an interesting issue that will need to be addressed in the future.

OPSCC and CUP frequently form histologically identifiable cystic node metastasis [12], [13]. Conversely, cystic node metastasis is often misdiagnosed as being a branchial cleft cyst [15]. In turn, it is not until surgical intervention followed by histopathological examination that the branchial cleft cyst can be proven to be cystic node metastasis. This is because both cystic node metastasis and branchial cleft cyst present radiographically as lateral cervical cysts, and because the sensitivity of cytopathological examination is poor in the detection of malignant cells in cystic fluid. In cystic node metastasis, the sensitivity of cytopathological diagnosis has been reported to be in the range of 33–50% [15], [21]. To find a solution regarding the differential diagnosis of these two entities, we first addressed the relationship between the primary site and radiographically identifiable cystic node metastasis on contrast-enhanced CT imaging. We found that radiographically identifiable cystic node metastasis was specific to OPSCC and CUP, and that the subsite of OPSCC with radiographically identifiable cystic node metastasis was either the palatine tonsil or the base of the tongue. Because the primary site specific to cystic node metastasis was identical to the primary site specific to HPV infection, we next investigated the relationship between HPV status and cystic node metastasis. We found that radiographically identifiable cystic node metastasis is more likely to be HPV-positive as compared with solid or necrotic node metastasis in OPSCC and CUP. These findings indicate that HPV positivity of the cystic fluid collected by FNA will help in making a definitive diagnosis, namely that the cyst of interest is malignant. In situ hybridization, which can be used in the detection of HPV DNA in cell blocks prepared from cystic fluid collection, seems inadequate for this purpose because of low cellularity. PCR, which is superior to in situ hybridization in sensitivity, is likely to serve more effectively for the detection of HPV DNA in cystic fluid collection. In the present study, we have demonstrated for the first time the feasibility of detecting HPV DNA using nested PCR in fluid collections aspirated from cystic node metastases. Collectively, we have shown that radiographically identifiable cystic node metastasis is most probably HPV-positive, and that PCR enables the detection of HPV DNA in fluid collection using FNA. These findings indicate that a lateral cervical cyst whose FNA fluid is HPV-positive is cystic node metastasis, but not a branchial cleft cyst.

On contrast-enhanced CT imaging, 6% of node-positive OPSCC and 11% of node-positive CUP showed cystic node metastasis. This prevalence of radiographically identifiable cystic node metastasis in OPSCC and CUP is relatively low, as compared with that of histologically identified cystic node metastasis. As much as 56 and 73% of node-positive cancers of the palatine tonsil and the base of the tongue, respectively, have been reported to form histologically identifiable cystic node metastasis [12]. This difference most probably reflects the limited ability of contrast-enhanced CT imaging to visualize a cystic lesion. It is difficult to differentiate a cystic lesion from a necrotic lesion, particularly when the lesion of interest is small. Moreover, even when a large node metastasis appears necrotic on contrast-enhanced CT imaging, FNA sometimes yields a fluid collection, indicating that radiographically the node appears to be necrotic but histologically it appears to be cystic.

In conclusion, we have established that the HPV status of the tumor remains unchanged after metastasis. In HPV-positive CUP, the occult primary tumor is most probably localized in the oropharynx, particularly either in the palatine tonsil or in the base of the tongue. HPV-positive OPSCC and CUP tend to form cystic node metastasis. PCR analysis of the FNA fluid enables nonsurgical detection of HPV DNA, which may facilitate the differential diagnosis of cystic node metastasis and the brachial cleft cyst.

## Supporting Information

Table S1 Characteristics of cancer of unknown primary in retrospective setting.
(xlsx)

Table S2 Characteristics of Cancer of Unknown Primary in Prospective Setting.
(XLSX)
