# Correction: Human Papillomavirus and Cystic Node Metastasis in Oropharyngeal Cancer and Cancer of Unknown Primary Origin

**DOI:** 10.1371/journal.pone.0116030

**Published:** 2014-12-15

**Authors:** The PLOS ONE Staff

There is an error in the title for Figure 1. The complete, correct Figure 1 title is: Radiographically identifiable (A) cystic, (B) necrotic, and (C) solid node metastasis defined by axial contrast-enhanced CT scans.

**Figure 1:**
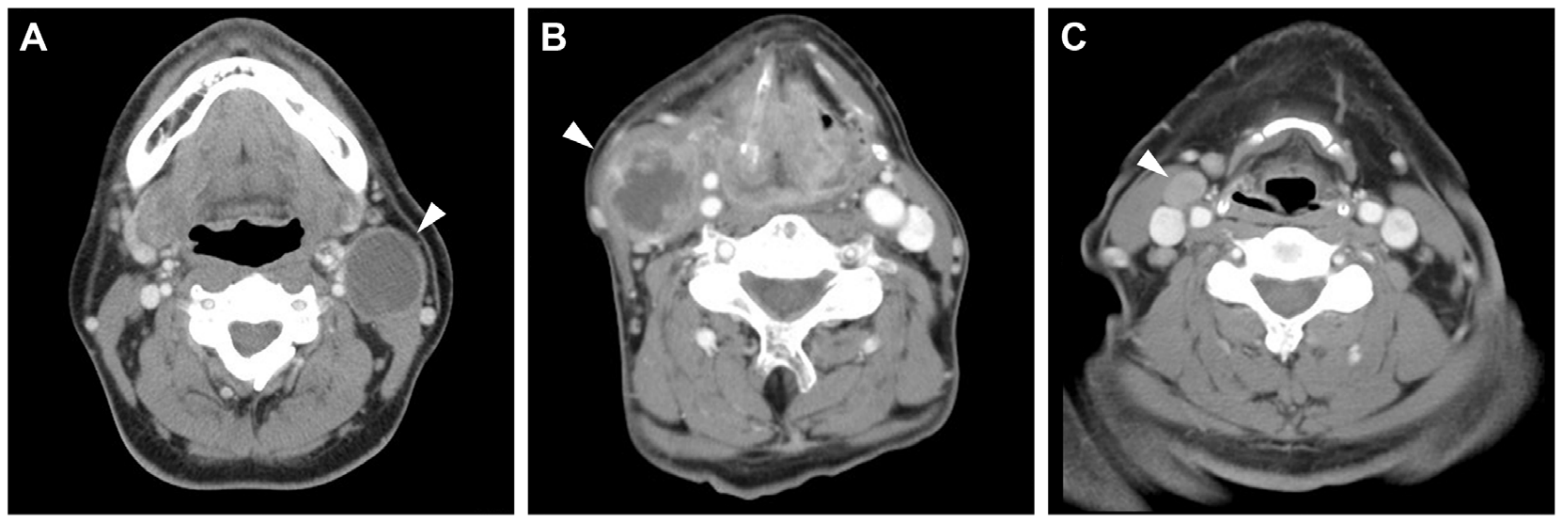
Radiographically identifiable (A) cystic, (B) necrotic, and (C) solid node metastasis defined by axial contrast-enhanced CT scans. Note a contrast-enhancing thin wall and homogeneous low-density content in the cystic node metastasis.
